# Prevalence, clinical presentations, and feto-maternal outcomes of eclampsia in a teaching hospital setting in Tigray region, Ethiopia: A five-year review

**DOI:** 10.1371/journal.pone.0332620

**Published:** 2025-10-08

**Authors:** Rahel Kidanemariam, Hale Teka, Habtom Tadesse, Mohamedawel Mohamedniguss Ebrahim, Ephrem Berhe, Bisrat Tesfay Abera, Marta Abrha, Hiluf Ebuy Abraha, Awol Yemane, Yibrah Berhe

**Affiliations:** 1 Department of Obstetrics and Gynecology, School of Medicine, College of Health Sciences, Mekellle University, Mekelle, Ethiopia; 2 Quality Assurance Office, College of Health Sciences, Mekellle University, Mekelle, Ethiopia; 3 Department of Internal Medicine, School of Medicine, College of Health Sciences, Mekellle University, Mekelle, Ethiopia; 4 Arnold School of Public Health, University of South Carolina, Columbia, South Carolina, United States of America; Delta State University, NIGERIA

## Abstract

**Background:**

Hypertensive disorders of pregnancy remain a leading cause of preventable maternal and perinatal mortality, particularly in low‑ and middle‑income countries (LMICs). Eclampsia, the most severe manifestation, is responsible for a disproportionate share of maternal deaths in sub‑Saharan Africa, yet contemporary data from Tigray, Ethiopia are scarce.

**Objective:**

To determine the prevalence, clinical presentation, and factors associated with adverse feto‑maternal outcomes among women managed for eclampsia at Ayder Comprehensive Specialized Hospital (ACSH), Tigray, Ethiopia, between 1 January 2017 and 31 December 2021.

**Methods:**

We conducted a retrospective cross‑sectional review of all women with a discharge diagnosis of eclampsia. A piloted extraction tool, adapted from recent studies, was used to abstract socio‑demographic, obstetric, clinical, laboratory, management, and outcome variables from medical charts. EpiData v4.6 was used for data entry, and data were analysed with Stata v16. Descriptive statistics summarised prevalence and presentation. Multivariate logistic regression identified independent predictors of (i) maternal end‑organ injury and (ii) perinatal death, reporting adjusted odds ratios (aOR) with 95% confidence intervals (CI). Model fitness was assessed with the Hosmer–Lemeshow test (p > 0.05).

**Results:**

Of 23,090 deliveries during the study period, 252 women (1.1%, 95% CI 1.0–1.2%) were diagnosed with eclampsia; 240 charts were analysed. The case‑fatality rate was 3.3% and perinatal mortality 20.1%. Antepartum eclampsia accounted for 63.8%, intrapartum 9.6%, and postpartum 26.7%. Headache (77.5%), visual disturbance (53.8%), and epigastric/right‑upper‑quadrant pain (46.3%) were the most frequent prodromal symptoms. Independent predictors of maternal end-organ injury were referral from another facility (aOR 4.9, 95% CI 1.8–13.9) and having perinatal death (aOR 2.7, 95% CI 1.2–6.1). Vaginal delivery (aOR 5.5, 95% CI 2.3–13.3), and pregnancies complicated with postpartum haemorrhage (aOR 3.2, 95% CI 1.2–8.3), acute respiratory distress syndrome (aOR 3.2, 95% CI 1.1–9.3), and dialysis‑requiring acute kidney injury (aOR 24.7, 95% CI 5.6–109.9) were independent predictors of perinatal death.

**Conclusions:**

Eclampsia prevalence at ACSH remains high and is associated with substantial maternal and perinatal mortality. Strengthening antenatal surveillance, streamlining referral pathways, and ensuring timely definitive delivery are critical to improving outcomes. Context-specific quality‑improvement initiatives should prioritise the prevention and aggressive management of hypertensive disorders of pregnancy.

## Introduction

Globally, approximately 260, 000 women died in 2023 from pregnancy-related causes—92% in LMICs [1]. Hypertensive disorders, led by pre‑eclampsia and eclampsia, account for 12–19% of these deaths [2]. These disorders, form a deadly triad with obstetric haemorrhage and sepsis, significantly impacting maternal and perinatal outcomes [3]. Between 2000 and 2023, the maternal mortality ratio (MMR, number of maternal deaths per 100 000 live births) dropped by about 40% worldwide. Despite global declines, progress in sub-Saharan Africa has stagnated, compounded by conflict and health‑system disruption [2]. The WHO report show that the sub-Saharan Africa alone contributed to the 70% (182,000 maternal deaths) of the global maternal mortality (1). In Ethiopia, the 2023 Maternal Mortality Estimation Inter‑Agency Group reported 267 deaths per 100 000 live births, with hypertensive disorders contributing 19% [4].

Eclampsia,defined as new‑onset generalised tonic‑clonic seizures in a woman with pre‑eclampsiacarries a case‑fatality rate up to 18.4% in West Africa [5]. Early warning signs (severe headache, visual disturbance, right‑upper‑quadrant pain) and biomarkers (angiogenic imbalance, severe proteinuria) permit risk stratification, but translation into routine practice in resource‑limited settings remains suboptimal [6]. The 2022 International Society for the Study of Hypertension in Pregnancy (ISSHP) guideline emphasises context‑adapted surveillance and magnesium‑sulfate prophylaxis, yet coverage gaps persist [7].

Clinically, eclampsia presents with warning signs such as severe headache, visual disturbances, epigastric pain, and elevated blood pressure [8]. Complications associated with eclampsia for the mother include cardiovascular and cerebrovascular diseases, liver and renal dysfunction, placental abruption, disseminated intravascular coagulation (DIC), and HELLP (Hemolysis, Elevated Liver enzymes, and Low Platelet count) syndrome [9]. Neonatal/fetal complications include oligohydramnios, non-reassuring fetal heart rate patterns, preterm birth, low birth weight, severe asphyxia, stillbirth, and intrapartum death [10,11]. Prompt recognition and management are crucial to prevent these complications, and standard management involves the use of magnesium sulfate for seizure control, antihypertensive therapy, and timely delivery of the fetus [8].

In Ethiopia, maternal mortality remains high, with hypertensive disorders, particularly eclampsia, contributing significantly to maternal and perinatal deaths [12–14]. Despite global efforts to reduce maternal mortality, eclampsia remains prevalent in Ethiopia, and comprehensive data on its clinical presentations and outcomes are scarce. Recent facility-based studies from Northwestern Ethiopia and Addis Ababa demonstrate eclampsia prevalences of 5.36% and 6.2%, respectively [15,16]. Gaps in early detection, inadequate antenatal care coverage, and delayed referrals exacerbate the burden of eclampsia in low-resource settings like Ethiopia [17].

Given the substantial impact of eclampsia on maternal and neonatal health, there is a critical need to understand its prevalence, clinical presentations, and outcomes within specific contexts. Evidence on predictors of adverse outcomes is inconsistent. Some studies implicate delayed presentation and multi‑organ dysfunction [18,19]; others highlight mode of delivery and quality of intrapartum care [10]. Generating locally relevant evidence is essential to tailor interventions. There is no accessible study that comprehensively quantified the burden, clinical spectrum, and determinants of poor outcomes among eclamptic women Tigray region.This study aimed to determine the prevalence, pattern of clinical presentation, and feto-maternal outcomes of mothers treated for eclampsia in Ayder Comprehensive Specialized Hospital, the second-largest teaching hospital in the Tigray region of Northern Ethiopia. The findings will provide valuable insights to inform healthcare strategies and interventions aimed at reducing the burden of eclampsia in similar low-resource settings.

## Methods

### Study desing

A retrospective cross-sectional study design was employed to examine the clinical profile, and maternal and neonatal outcomes of women diagnosed with eclampsia from January 1, 2017 – December 31, 2021, at Ayder Comprehensive Specialized Hospital. This was a review of patient records of antepartum, intrapartum, and postpartum visits.

### Study setting

Ayder Comprehensive Specialized Hospital is the largest referral and teaching hospital in Northern Ethiopia, serving a catchment area of more than nine million people and handling an annual patient volume of 300,000 (8). It accepts direct obstetric patient visits, including low-risk mothers, and functions as a high-risk referral hospital for the entire Tigray region, neighbouring districts of Amhara, and parts of Afar regional state. The number of obstetric patients has been steadily increasing, and the hospital now conducts an average of 5000 obstetric deliveries per year. There are two separate intensive care units for adult patients, namely Surgical and Medical Intensive Care Units (ICUs), as well as a Medical ICU. Since there is no separate Obstetric High Dependency Unit (HDU) or Obstetric ICU, critical obstetric patients are admitted either to the surgical ICU or the Medical ICU, depending on their condition. Eclamptic patients are primarily treated in the labour and maternity wards. Due to resource limitations, critical obstetric patients are admitted to ICUs only if mechanical ventilation is necessary. During the study period, Ayder was the only hospital in the region equipped with an ICU. As a result, most eclampsia cases from across health facilities were referred to Ayder Comprehensive Specialised Hospital due to concerns about complications requiring ICU admission. The hospital adheres to a policy of brief induction in eclamptic mothers, provided that the cervix is favourable and the mother is stable with seizure optimally controlled.

### Study population

The study population were women in antepartum, intrapartum, or postpartum periods diagnosed with eclampsia at Ayder Comprehensive Specialized Hospital during the study period, January 1, 2017 – December 31, 2021.

**Eligibility Criteria: –** Women who were diagnosed and treated for eclampsia during the study period were included in the study, while mothers with incomplete chart records or seizures due to other causes (e.g., epilepsy, meningitis, cerebrovascular accidents etc) were excluded.

### Data collection procedure and tools

A data‑abstraction instrument was first developed from the literature review (3,7,8,21) by the principal investigator. The draft tool was circulated to the full research team for critique; their suggestions were incorporated, and the instrument was adapted to the local context, yielding the final version used for data collection. Logbooks from the labour ward, operating theatre, emergency outpatient department, gynaecology ward, and surgical and adult medical ICUs were screened to identify cases of eclampsia, after which the corresponding medical records were retrieved from the archives. Fifteen trained physicians then reviewed each case, recording clinical and laboratory parameters as well as maternal and neonatal outcomes with the finalised tool. The questionnaire captured sociodemographic characteristics, obstetric history, clinical presentation, laboratory findings, management details, and outcomes.

### Data quality control

The data abstraction’s clarity was tested through a pretest conducted on 5% of the sample size. One-day training was provided to data collectors and supervisors regarding the objective of the study, data collection methods, and significance of the study. Fifteen trained physicians extracted data after a one‑day workshop; inter‑rater agreement (κ = 0.92) was confirmed on 5% of records. Supervisors monitored the data collection process, and necessary corrections were made accordingly.

### Operation definitions

For this study, we have used the following criteria to define end-organ damage for each organ system of the body.

- Central nervous dysfunction: Coma, temporary or permanent blindness
- Renal insufficiency: renal insufficiency (creatinine > 1.2), oliguria, or anuria
- Hemolysis, Elevated Liver Enzymes, and Low Platelete count (HELLP) Syndrome: platelet count < 100,000, mmol/L, aspartate transaminase (AST) > 62, alanine transaminase (ALT) > 64, and HDL > / = 600 U/L
- Liver dysfunction: Liver function test derangements twice the above normal (i.e., AST > 62, and ALT > 64). In hospitals’ laboratories, the upper normal for AST and ALT is 31 and 32, respectively.
- Respiratory dysfunction: Clinically evident pulmonary edema
- Hematologic dysfunction: Obstetric haemorrhage requiring transfusion.

### Data processing and analysis

EpiData 4.6 was used for data entry. After ensuring completeness, the collected data were exported to Stata version 16 for further data cleaning and data analysis. During data cleaning in STATA, missing data on key variables were addressed by repeatedly reviewing patient charts and other medical records. Descriptive statistics, including frequencies, percentages, mean (M), and standard deviation (SD), were employed to summarize the data. A binary logistic regression model was fitted to control for confounding variables and identify independent predictors. Variables with p-value below 0.250 during bivariate analysis were selected for multivariable binary logistic regression model. The odds ratio, along with its 95% confidence interval (CI) and p-value, was reported to indicate the magnitude, direction, and significance of the association. In the multivariable analysis, p-values less than 0.05 were considered statistically significant. Hosmer-Lemeshow test p-value of 0.05 or above was used to consider the multivariable models are good-fit for the data. Variance Inflation Factor (VIF) values less than 10 were used as the cutoff point to assume the absence of multicollinearity.

### Ethical considerations

Ethical approval was obtained from the Institutional Review Board (IRB) (MU- IRB 1950/ 2022) of Mekelle University, College of Health Sciences. This was part of a large maternal near-miss and mortality research project in ACSH. Permission for data collection was obtained from the Chief Clinical Director (CCD) of ACSH. Informed consent was waived by the IRB as we used secondary data. Patient identifiers were fully anonymised. Patient charts were accessed and reviewed from May 1, 2022 – June 30, 2022.

## Result

### Socio-demographic characteristics

**Fig 1:**
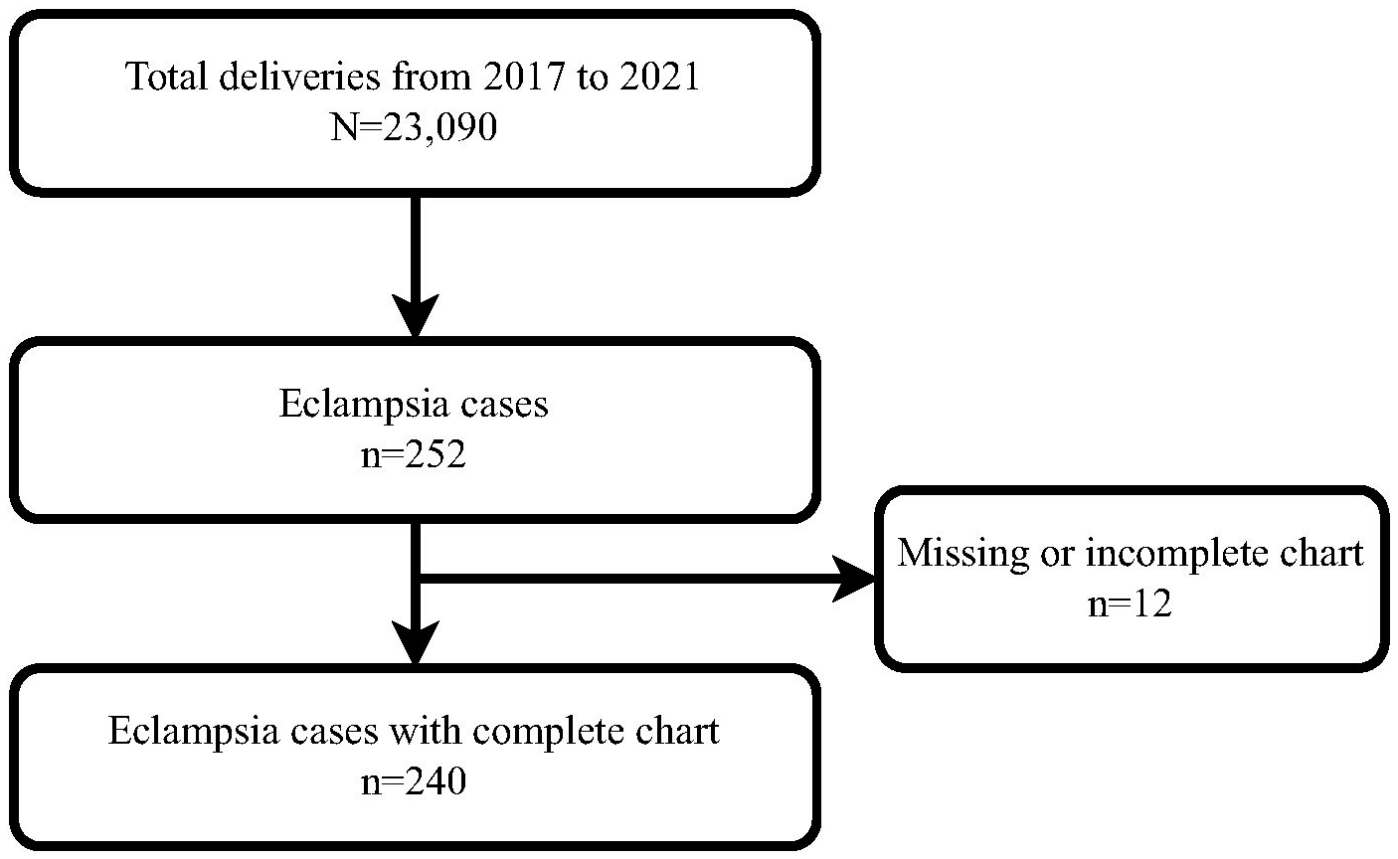
Flow chart of total deliveries and eclampsia cases in Ayder Comprehensive Specialized Hospital from January 1, 2017 to December 31, 2021.

Among the 20,090 women who delivered during the study period, 252 cases of eclampsia were recorded, giving a prevalence of 11.0 per 1,000 deliveries (1.1% [95% CI 1.0%–1.2%]). Twelve patient charts were excluded because essential variables were missing (Fig 1).

Of the 240 records analyzed, the case‑fatality rate was 3.3%. Most patients, 188 (78.3%), were 20–35 years old; the mean age was 24.6 years (SD = 5.8), and 16.7% were teemagers. Overall, 158 participants (65.8%) lived in rural areas, and 210 (87.5%) were referred from another facility, predominantly other hospitals (Table 1).

**Table 1:** Socio-demographic characteristics of eclamptic women delivered at Ayder Comprehensive Specialized Hospital, Mekelle, Ethiopia 2017–2021 (n = 240).

***Sociodemographic and referral characteristics***	***Frequency (n = 240)***	***Percent [95% CI]***
Age category		
<20	40	16.7[12.4,22.0]
20-35	188	78.3[72.6,83.0]
>35	12	5.0[28.5,86.2]
Address		
Rural	158	65.8[59.6,71.6]
Urban	82	34.2[28.4,40.4]
Ethnicity		
Tigray	207	86.3[81.2,90.0]
Afar	28	11.7[16.4,81.6]
Amhara	5	2.1[0.86,4.9]
Referral type		
Self-referred*	30	12.5[8.9,17.3]
With referral paper	210	87.5[82.6,91.1]
Referring institution (n = 210)		
Health center	69	32.9[26.8,39.5]
Hospital	124	59.0[52.2,65.5]
Private	15	7.1[4.3,11.6]
Not specified	2	1.0[0.2,0.4]

*Self-referral in this study stands for mothers who had antenatal care follow-up at Ayder Comprehensive Specialised Hospital and who developed eclampsia in the course.

### Obstetrical history of current pregnancy among the eclamptic mother

Nearly two‑thirds of the participants were primiparous; 63 (26.3%) were multiparous and 24 (10.0%) were grand‑multiparous. More than three‑quarters—191 (79.6%)—received regular antenatal care, and 10.4% had a history of at least one abortion. In the current pregnancy, almost all women (237; 98.8%) received magnesium sulfate, while hydralazine, nifedipine, and diazepam were given to 51.3%, 30.4%, and 15.4% of patients, respectively. Antibiotics were prescribed for 222 participants (92.5%), most commonly the combination of ceftriaxone and metronidazole (201; 90.5%) (Table 2).

**Table 2:** Current obstetrical history of eclamptic mothers delivered at Ayder comprehensive Specialized Hospital, Mekelle, Ethiopia 2017–2021 (n = 240).

Features	Frequency (n = 240)	Percent (95% CI)
Antenatal care visit		
Yes	191	79.6[73.97,84.25]
No	49	20.4[15.75,26.03]
ANC providing facility(n = 191)		
Health center	133	69.6[62.68,75.79]
Hospital	46	24.1[18.5,30.72]
Private	10	5.2[2.82,9.51]
Not specified	2	1.0[0.26,4.14]
Parity		
Nullipara	2	0.8[0.21,3.30]
Primipara	151	62.9[56.6,68.8]
Multipara	63	26.3[21.0,32.2]
Grand multipara	24	10.0[6.8,14.5]
GA category(n = 121)		
<28	9	7.4[3.88,13.79]
28-33 + 6	34	28.1[20.72,36.88]
34-36 + 6	35	28.9[21.46,37.75]
37-41 + 6	38	31.4[23.67,40.33]
42+	5	4.1[1.71,9.66]
Time of eclampsia diagnosis		
Antepartum	153	63.8[57.43,69.63]
Intrapartum	23	9.6[6.43,14.05]
Postpartum	64	26.7[21.42,32.66]
Drugs given during the current pregnancy		
Magnesium sulphate	238	98.8[96.17,99.6]
Hydralazine	123	51.3[44.89,57.56]
Nifedipine	73	30.4[24.89,36.57]
Diazepam	37	15.4[11.35,20.60]
Methyldopa	22	9.2[6.09,13.57]
Phenytoin	19	7.9[5.09,12.11]

*ANC: Antenatal Care; GA: Gestational age*

### Clinical presentation of the mothers with eclampsia

More than one‑quarter of the eclamptic patients—27.5%—presented with a systolic blood pressure > 160 mm Hg, and 18.3% had a diastolic pressure > 110 mm Hg. Forty‑nine women (20.4%) were normotensive (BP < 140/90 mm Hg), and approximately one‑third (34.6%) exhibited proteinuria of +3 or higher. Three‑quarters of the patients experienced their first seizure at home, and 114 (47.5%) reached our hospital more than 12 hours after that initial episode.

Almost half of the participants,119 (49.6%),delivered vaginally, while 97 (40.4%) underwent caesarean section. The principal indications for caesarean delivery were eclampsia with an unfavourable Bishop score (23.7%), a non‑reassuring fetal heart‑rate pattern (15.5%), twin pregnancy (15.5%), and poor progress of labour (8.2%). Twelve mothers had abortive outcomes (11 expelled vaginally and 1 via hysterotomy) (Table 3).

**Table 3:** clinical features and delivery history of eclamptic mothers who delivered at Ayder Comprehensive Specialized Hospital, Mekelle, Ethiopia *2017–2021* (n = 240).

Features	Frequency (n = 240)	Percent (95% CI)
Headache	186	77.5(71.74,82.37)
Visual disturbance	129	53.8(47.37,60.01)
Epigastric/Right upper quadrant pain	111	46.3(39.99,52.63)
Vomiting	54	22.5(17.63,28.26)
Coma	42	17.5(13.17,22.88)
Place of seizure		
Home	166	69.2[62.99,74.72]
Health facility	74	30.8[25.28,37.01]
First seizure episode to arrival at hospital (hours)		
Within 12hrs.	126	52.5[46.13,58.79]
After 12 hrs.	114	47.5[41.21,53.87]
Proteinuria	156	65.0[58.71,70.81]
SBP(above 160)	66	27.5[22.19,33.54]
DBP (above 110)	44	18.33[13.90,23.78]
ALT > 64 (N = 193)	121	62.7[55.60,69.28]
AST >=62(N = 215)	101	47.0[40.35,53.72]
LDH >=600 (N = 39)	19	48.7[33,64.70]
Mode of delivery		
Spontaneous Vaginal delivery	117	48.8[42.4,55.1]
Caesarean delivery	97	40.4[34.4,46.8]
Forceps	9	3.8[2.0,7.1]
Vacuum	5	2.1[0.9,4.9]
Hysterotomy	1	0.4[0.06,2.9]
Expelled	11	4.6[2.6,8.1]

*ALT: Alanine transaminase; AST: Aspartate aminotransferase; DBP: Diastolic Blood Pressure; LDH: Lactate Dehydrogenase; SBP: Systolic Blood Pressure*

### Maternal and fetal outcomes of the eclamptic patients

Among the eclamptic patients, 131 (54.6%) developed end‑organ damage, 53 (22.1%) experienced HELLP syndrome, and 26 (10.8%) suffered postpartum haemorrhage. Intensive‑care admission was required for 44 women (18.3%), and acute kidney injury occurred in 57 (23.8%). Eight maternal deaths were recorded; all affected women had been referred from other facilities. Multi‑organ failure was the immediate cause of death in three cases, while amniotic‑fluid embolism, brain death, magnesium‑sulfate toxicity, intracranial haemorrhage, and cardiac arrest secondary to hypoxic‑ischaemic injury accounted for the remaining five (Table 4).

**Table 4:** Outcome of eclamptic mothers delivered at Ayder Comprehensive Specialized Hospital, Mekelle, Ethiopia 2022 (N = 240).

	Frequency(n = 240)	Percent
Complication		
Abruption	21	8.8[5.8,13.1]
Postpartum Hemorrhage	26	10.8[7.5,15.5]
Deep Intravascular Coagulation	10	4.2[2.3,7.6]
HELLP syndrome	53	22.1[17.3,27.8]
End organ injury	131	54.6[48.2,60.8]
ARDS	20	8.3[5.4,12.6]
Coma	42	17.5[13.2,22.9]
Liver injury	93	38.8[32.8,45.1]
AKI	57	23.8[18.8,29.6]
Dialysis(n = 57)	12	21.1[4.1,10.6]
ICU admission	44	18.3[13.9,23.8]
Blindness	7	2.9[1.4,6.0]
Death	8	3.3[1.7,6.6]

*ARDS: Acute Respiratory Distress Syndrome; HELLP Syndrome: HE – Hemolysis, EL- Elevated Liver Enzymes, LP – Low Platelet Count; ICU – Intensive Care Unit.*

### Fetus related characteristics

Of the 240 women studied, 22.6% delivered stillborn infants. Among the 202 live births (77.4%), 46 neonates (17.7%) had a 5‑minute Apgar score below 6, and almost half (49.8%) were low birth‑weight at delivery (Table 5).

**Table 5:** Perinatal outcome of eclamptic mothers delivered at Ayder Comprehensive Specialized Hospital, Mekelle, Ethiopia *2017-2021* (n = 240).

	Frequency(n = 261)	Percent
Sex of the fetus(n = 261)		
Male	115	44.1
Female	106	40.6
Undocumented	40	15.3
Fetal outcome (n = 261)		
Alive	202	77.39
Stillbirths	59	22.61
Number of fetus(n = 261)		
Singleton	219	91.3
Twin	21	8.8
First Minute APGAR Score (n = 261)		
0	35	13.4
1-6	52	19.9
7-10	107	41
Not documented	67	25.7
Fifth Minute APGAR score (n = 261)		
0	35	13.4
1-6	11	4.2
7-10	148	56.7
Not documented	67	25.7
Birth weight category		
<1500	37	14.2
1500-2499	93	35.6
2500-3999	76	29.1
>4000	5	1.9
Undocumented	50	19.2

### End organ injury and its predictors

End‑organ injury was more common among women referred from other facilities (59.5%) than among those who self‑presented (20%). Similarly, women whose first seizure occurred at home faced a 20% greater risk of end‑organ damage. To explore determinants of this complication, we built a multivariable logistic‑regression model incorporating seven candidate predictors: maternal age, length of hospital stay, referral type, seizure location, fetal outcome, postpartum haemorrhage, and vomiting. In bivariable analyses, five variables—length of stay, referral type, seizure location, fetal outcome, and vomiting—were significantly associated with end‑organ injury. In the multivariable model, only two factors remained independent predictors: referral from another facility (aOR≈ 5.0) and perinatal death (aOR≈ 2.7). The model showed an acceptable fit (Hosmer–Lemeshow test, p = 0.588) (Table 6).

**Table 6:** Factors associated with end organ damage in eclamptic mothers delivered at Ayder Comprehensive Specialized Hospital, Mekelle, Ethiopia *2017-2021* (n = 240).

	*End organ injury* *, n (row %)*		*Odds Ratio*	
***Variables***	***No***	***Yes***	***COR [95% CI]***	***AOR [95% CI]***
Age (years), M (SD)	23.9 (5.4)	25.2 (6.0)	1.04 [0.99, 1.09]	1.04[0.99,1.10]
Hospital Stay, Median (IQR)	5 (3)	6 (5)	1.12 [1.04, 1.20] **	1.06[0.99,1.12]
Referral type				
Self-referral	24 (80.0)	6(20.0)	1	1
Had referral paper	85(40.5)	125(59.5)	5.88 [2.31, 15.00] ***	4.93 [1.75, 13.87] **
Seizure place				
Health facility	45(60.8)	29(39.2)	1	1
Home	64(38.6)	102(61.4)	2.47 [1.41, 4.33] **	1.78 [0.94,3.37]
Fetal outcome				
Alive	96(53.9)	82(46.1)	1	1
Perinatal death	10(16.9)	49(83.1)	4.68 [2.20, 9.94] ***	2.72[1.22, 6.10] *
Postpartum Haemorrhage				
Yes	7(26.9)	19(73.1)	2.47 [1.00, 6.12]	2.03 [0.76, 5.43]
No	102(47.7)	112(52.3)	1	1
Vomiting				
Yes	18(33.3)	36(66.7)	1.92 [1.02, 3.61] *	1.42 [0.69, 2.95]
No	91(48.9)	95(51.1)		

*p < 0.05 **p < 0.01 ***p < 0.001

### Perinatal death and its predictors

Perinatal mortality was 14% higher among mothers whose first seizure occurred at home than among those whose first seizure occurred in a health facility. The risk of perinatal death was more than threefold greater after vaginal delivery than after caesarean section. Likewise, perinatal deaths were markedly more frequent in women who experienced postpartum haemorrhage, developed acute respiratory distress syndrome (ARDS), or required dialysis.

Multivariable logistic‑regression analysis identified four independent predictors of perinatal death. Vaginal delivery conferred an adjusted odds ratio (AOR) exceeding 5 compared with caesarean delivery, while postpartum haemorrhage and ARDS were associated with AORs of 3.2. Acute kidney injury severe enough to necessitate dialysis was the strongest predictor: 13 of the 16 women who underwent dialysis (81.2%) sustained a perinatal loss, yielding an AOR of 24.7. The model exhibited a satisfactory fit to the data (Hosmer–Lemeshow test, p = 0.252; Table 7).

**Table 7:** Factors associated with poor fetal outcome (still birth) at delivery in eclamptic mothers who delivered at Ayder Comprehensive Specialized Hospital, Mekelle, Ethiopia *2017–2021* (n = 240).

	*Perinatal death, n (row %)*		*Odds Ratio*	
*Variables*	*No*	*Yes*	*COR [95% CI]*	*AOR [95% CI]*
Seizure place				
Health facility	63(85.1)	11(14.8)	1	1
Home	118(71.1)	48(28.9)	2.33[1.13,4.80] *	2.24 [0.98, 5.12]
Mode of delivery				
Vaginal delivery	92(64.8)	50(35.2)	5.37[2.50,11.57] ***	5.52[2.29,13.30] ***
Cesarean/Hysterotomy	89(90.8)	9(9.2)	1	1
PPH				
Yes	14(53.9)	12(46.1)	3.05[1.32,7.03] **	3.18[1.21,8.31] *
No	167(78.0)	47(22.0)	1	1
ARDS				
Yes	10(50.0)	10(50.0)	3.49[1.37,8.87] **	3.18[1.08,9.34] *
No	171(77.7)	49(22.3)	1	1
Dialysis				
Yes	3(18.8)	13(81.2)	16.77[4.59,61.32] ***	24.7 [5.55, 109.85] ***
No	178(79.5)	46(20.5)	1	1

*p < 0.05 **p < 0.01 ***p < 0.001

## Discussion

The aim of this study was to determine the prevalence, pattern of clinical presentation and feto-maternal outcomes of eclampsia in a tertiary care hospital in northern Ethiopia over a five-year period. The prevalence of eclampsia was 1.1 (95% CI, 1.0–1.2), maternal mortality rate was 3.3% and perinatal mortality rate was 20.1%. Most cases occurred in women of rural residence and primipara. The most common symptoms included headache, visual disturbances and epigastric/right upper quadrant pain.

The 1.1% prevalence of eclampsia we report is five to ten times higher than estimates from high‑income countries (<0.2%) [20], but is lower than prevalences in other Ethiopian regions [15,16] . The relatively low rate of eclampsia in Tigray, compared with findings from studies in other regions in Ethiopia, may be attributed to the relatively strong and functional community health extension worker system, which facilitates earlier referral of mothers with hypertensive disorders and the provision of prophylactic management. To the contrary, the persistent excess in low‑resource settings likely reflects gaps in antenatal risk screening, limited prophylactic use of low-dose aspirin, and delayed escalation of severe pre‑eclampsia [21]. To this end high‑quality blood‑pressure and proteinuria assessment at the primary‑care level remains a critical,and achievable priority in the Ethiopian essential maternal‑health package. However, comparing eclampsia rates between low-income and high-income countries is inherently challenging. In high-income settings, where institutional delivery rates are high, reported eclampsia rates are more likely to reflect the true population prevalence. In contrast, in low-income countries with a high proportion of home births, institutional rates may underestimate the actual burden of eclampsia in the community.

Nearly two‑thirds of cases presented before the onset of labour, a distribution consistent with a review by Beyu et al [22] which showed the proportion of antepartum eclampsia to be 68.7% in Ghana. Earlier seizure onset in our context may stem from limited ANC contacts, under‑recognition of prodromal symptoms, and long referral distances. Strengthening community education on warning signs and introducing pre‑referral magnesium‑sulfate loading could shift the spectrum towards milder disease on arrival and reduce antepartum seizures.

Maternal end‑organ injury complicated more than half of the cases and was five‑fold more likely among women referred from other facilities. Similar associations between inter‑facility transfer and multi‑organ dysfunction have been documented in Ghana [23], suggesting that transport delays, sub-optimal stabilisation, or shortages of magnesium sulfate at lower‑level centres amplify systemic damage. In the present study, Eclampsia was also associated with a high case fatality rate. Establishing obstetric high‑dependency units and standardising referral algorithms with mandatory magnesium‑sulfate loading and antihypertensive therapy are pragmatic interventions with the potential to curb organ failure and death.

Perinatal mortality far exceeded a study from Kenya, which reported 9.4% [24] and was quadrupled in vaginal versus caesarean deliveries. Although caesarean section carries operative risks, expedited surgical delivery shortens fetal exposure to hypoxia during uncontrolled seizures, explaining the survival advantage observed in meta‑analyses of severe pre‑eclampsia/eclampsia before 34 weeks [24]. The association of poor perinatal outcomewith postpartum haemorrhage, ARDS, and dialysis highlight the interdependence of maternal physiology and neonatal survival: when the mother fails, so too does the fetus [25]. Scaling up timely caesarean capacity, ensuring blood‑bank readiness, and integrating neonatal resuscitation teams into obstetric emergencies are immediate programmatic implications. Moreover, the low rate of instrumental delivery (5.9%), which is indicated to shorten the second stage of labor in all eclamptic mothers, is concerning. This may be related to limited skills training and simulation-based practice in instrumental delivery emphasizing the need to scale up the training of this skill set.

### Strengths and limitations

The strength of this study lies in the comprehensive evaluation over five years in a tertiary hospital, which provides valuable data for the region. The large sample size increases the reliability of the results. However, the retrospective design may lead to information bias due to incomplete records. To this end, important variables such as seizure while on treatment, decision to delivery time, and reasons surrounding for the choice of mode of delivery are missing. In addition, the generalizability of the results to the wider population may be limited by the fact that this is a single center study. Further research is needed to identify barriers to early presentation and access to care for women with eclampsia. Qualitative studies examining pregnant women's perceptions and knowledge of hypertensive disorders could provide insights to improve education programs. In addition, studies evaluating the effectiveness of interventions to reduce the incidence and improve outcomes of eclampsia in low-resource settings are warranted.

## Conclusion

Despite the efforts made in Ethiopia to combat eclampsia, its prevalence remains high and is associated with significant maternal and perinatal mortality and morbidity. The high prevalence emphasizes the need for increased attention to antenatal care, which should be carried out effectively at all levels of the healthcare system. Early detection and diagnosis of eclampsia is essential to reduce both maternal and perinatal morbidity and mortality. Interventions should focus on improving access to quality care for pregnant mothers within the health system. Concerted efforts are needed to improve access to quality prenatal care, promote early detection and diagnosis, improve access to and optimisation of referral support, and increase institutional awareness of the importance of reducing maternal mortality rates.

## Supporting information

S1 Data Dataset. (XLS)
